# Biomarkers and Mechanisms of Cardiovascular Susceptibility and Resilience to Post-Traumatic Stress Disorder

**DOI:** 10.31083/RCM44081

**Published:** 2025-11-26

**Authors:** Eugenia B. Manukhina, Maryrita K. Mallet, Vadim E. Tseilikman, Marina V. Kondashevskaya, Olga P. Budanova, H. Fred Downey, Robert T. Mallet

**Affiliations:** ^1^Laboratory for Regulatory Mechanisms of Stress and Adaptation, Institute of General Pathology and Pathophysiology, 125315 Moscow, Russia; ^2^Department of Physiology and Anatomy, University of North Texas Health Science Center, Fort Worth, TX 76107, USA; ^3^School of Basic Medicine, Chelyabinsk State University, 454001 Chelyabinsk, Russia; ^4^Center “Biomedical Technology”, School of Medical Biology, South Ural State University, 454080 Chelyabinsk, Russia; ^5^Zelman School of Medicine, Novosibirsk National Research State University, 630090 Novosibirsk, Russia; ^6^Laboratory of Cell Pathology, Avtsyn Research Institute of Human Morphology of the Petrovsky National Research Center of Surgery, 117418 Moscow, Russia

**Keywords:** biomarkers, cardiovascular system, catecholamines, cytokines, glucocorticoids, inflammation, myocardial injury, post-traumatic stress disorder, oxidative stress, psychotherapy

## Abstract

Post-traumatic stress disorder (PTSD), which develops in susceptible individuals after life-threatening or traumatizing events, manifests as a heightened anxiety and startle reflex, disordered sleep, nightmares, flashbacks, and avoidance of triggers. Moreover, PTSD is a predictor and independent risk factor of numerous cardiovascular comorbidities, including stroke, myocardial infarction, coronary atherosclerosis, and atrial fibrillation. Compounding the direct detrimental effects of PTSD on the cardiovascular system, this condition provokes classical cardiovascular risk factors, including high cholesterol and triglycerides, platelet hyperaggregation, endothelial dysfunction, hypertension, and sympathetic hyperactivation. Although most people who have experienced traumatic events do not develop PTSD and are considered PTSD resilient, a substantial minority experience persistent cardiovascular comorbidities. Experimental and clinical studies have revealed a myriad of biomarkers and/or mediators of PTSD susceptibility and resilience, including pro- and anti-inflammatory cytokines, oxidized proteins and lipids, antioxidants, troponin, catecholamines and their metabolites, glucocorticoids, and pro-coagulation factors. The use of biomarkers to predict cardiovascular susceptibility or resilience to PTSD may stratify the risk of a patient developing cardiovascular complications following severe stress. Indeed, since many PTSD biomarkers either inflict or attenuate cardiovascular damage, these biomarkers can be applied to monitor the efficacy of exercise, dietary modifications, and other interventions to enhance cardiovascular resilience and, thereby, restrict the detrimental effects of PTSD on the cardiovascular system. Biomarker-informed therapy is a promising strategy to minimize the risk and impact of cardiovascular diseases in individuals with PTSD.

## 1. Introduction

Post-traumatic stress disorder (PTSD) is a severe psychiatric condition that afflicts survivors or witnesses of traumatizing, generally life-threatening events, including disasters, abuse, adverse childhood experiences, military combat, violent crime, motor vehicle accidents, or death of loved ones [1]. Worldwide, the lifetime prevalence of PTSD is approximately 5.6%, or over 450 million individuals [2]. The constellation of PTSD symptoms includes heightened anxiety, hypervigilance, exaggerated startle reflex, disordered sleep, nightmares, flashbacks, and avoidance of flashback triggers [3, 4].

Most individuals possess psychological resilience, defined by the American Psychological Association as “*the process and outcome of successfully adapting to difficult or challenging life experiences, especially through mental, emotional, and behavioral flexibility and adjustment to external and internal demands*” [5]. The resilient individual adapts to stress in a manner that limits or prevents PTSD after traumatic events [6, 7]. Approximately 50–84% of individuals experience traumatizing events over their lifetime. Although resilient individuals recover from the mental and physiological responses to stress, typically within 1–4 weeks [8], approximately 10–13% develop PTSD [8, 9, 10]. The likelihood of developing PTSD depends heavily on the type of trauma; for example, the 2001 Australian National Survey of Mental Health and Well-Being revealed 49% of rape victims developed PTSD, vs. 32% of physical assault victims, 16.8% of serious accident victims, and 3.8% of survivors of natural disasters [11]. Approximately one-third of adult survivors of childhood neglect and sexual and physical abuse developed PTSD, while two-thirds of survivors displayed resilience that persisted into adulthood [12].

PTSD frequently manifests as physical disabilities and internal organ dysfunction [13], and the cardiovascular system is a primary PTSD target. Risk of hypertension, dyslipidemia, and type 2 diabetes, the cardiovascular disease (CVD) most closely associated with PTSD, parallels the severity of PTSD symptoms [11, 14]. PTSD is recognized as an independent risk factor, predictor, and even a cause of cardiovascular and related diseases [1, 14, 15, 16, 17], including cardiac arrhythmias, coronary artery disease (CAD), myocardial infarction, hypertension, stroke, venous thromboembolism, and diabetes [18]. PTSD exacerbates CVD and may precipitate sudden cardiac death [14, 15, 19, 20]. Meta-analyses of large clinical studies, adjusted for clinical depression and demographic, clinical, and psychosocial factors, showed PTSD to be associated with a 27% increase in cardiovascular events or cardiac-specific mortality [21, 22], including a 47% increased risk of heart failure [23] and a 55–61% increase of CAD [21, 22, 24]. In experimental studies, 35–40% of rats exposed to traumatizing events developed PTSD-like behaviors and had evidence of CVD, yet the majority were resilient [25, 26]. Thus, although most people and animals adapt to severe stress and, thereby, avoid its sustained, adverse psychiatric and cardiovascular consequences, a sizeable minority do not.

Low resistance to causative factors or the absence of protective factors predispose to PTSD-induced pathology. Indeed, identification of factors or biomarkers of PTSD vulnerability vs. resistance is an active research field. Vulnerability biomarkers can be early indicators of heightened risk of PTSD-induced CVD; thus, increases in these biomarkers may herald impending PTSD and its associated CVD. Conversely, individuals with biomarkers of PTSD resilience may be well-suited for stressful or dangerous endeavors, e.g., space flight or military combat. Moreover, in persons preparing for such endeavors, physical exercise, psychiatric and/or pharmacological interventions might augment psychological resilience and, thereby, lower the risk of subsequent PTSD-related CVD.

## 2. Association of PTSD With CVD Risk Markers

PTSD provokes the emergence and hastens the development of CVD risk factors [1, 14, 15, 16, 17], which portend major adverse cardiovascular events, including stroke, myocardial infarction, heart failure, and cardiovascular death. Due to its association with CVD risk factors, PTSD was estimated to increase the odds of adverse cardiovascular outcomes by 69% [21] and CAD by 55% [24]. Thus, special clinical attention to CVD risk factors in people exposed to trauma and to PTSD patients is warranted, and reductions of these factors may lower the risk of adverse cardiovascular events [14]. The CVD risk markers often seen in PTSD patients include decreased heart rate variability (HRV), an indication of decreased parasympathetic activity, and myocardial electrical instability, which raises the risk of ventricular arrhythmias and sudden cardiac death [27, 28].

Patients with PTSD show increased sympathetic activity and autonomic nervous system dysfunction [29, 30, 31], which directly correlates with the severity of PTSD symptoms [32]. When exposed to mental stressors, PTSD patients demonstrated heightened perceptions of and responses to threats. The resultant sympathetic hyperactivation elicited a cascade of massive catecholamine release and overproduction of cardiotoxic reactive oxygen species and pro-inflammatory cytokines [16, 29, 31]. Indeed, a meta-analysis of 54 clinical studies of PTSD patients revealed heightened inflammation with elevated inflammatory biomarkers, e.g., C-reactive protein, interleukin (IL)-6, and tumor necrosis factor-alpha (TNF-α) [33].

Unhealthy behaviors, including sedentary lifestyle [34], poor diet [35], smoking [36], alcohol [37], or drug abuse [38], and noncompliance with prescribed treatment [39], are important CVD risk factors. Failure to follow recommended and prescribed methods of primary and secondary CVD prevention exacerbates PTSD-related damage to the cardiovascular system [16, 19]. These modifiable lifestyle factors are considered CVD risk biomarkers in PTSD patients [40].

The risk of PTSD may be genetically predetermined, at least in part [41]. A causative relationship between PTSD and hypertension was confirmed by Mendelian randomization of genome-wide association studies [42, 43]. Studies of twins demonstrated 40–60% heritability of PTSD susceptibility [44]. To identify marker genes, Pollard *et al*. [45] examined 106 studies reporting one or more polymorphic variants in 87 candidate PTSD risk genes. Of those genes, 36 overlapped and were significantly associated with independent CVD risk genes, with which they shared proinflammatory signaling mechanisms. Genome-wide association analyses revealed common genetic factors for PTSD and hypertension, heart failure, and CAD [44]. These genetic correlations paralleled CVD risk factors, including insomnia, increased waist-to-hip ratio, smoking, alcohol dependence, and the inflammatory markers IL-6 and C-reactive protein. Mendelian randomization revealed a strong causal effect of PTSD on CAD and a weaker causal effect on hypertension and heart failure [45].

## 3. Myocardial Injury and Resilience in PTSD

As noted above, severe stress initiates the pathogenesis of PTSD and its cardiovascular sequelae. The cardiac effects of severe stress were described initially by Da Costa in 1871 in American Civil War veterans [46]. Terming the disorder “soldier’s heart”, Da Costa identified palpitations, fatigue, dyspnea, chest pain, sighing, dizziness, faintness, apprehensiveness, headache, paresthesia, weakness, trembling, insomnia, and unhappiness among its symptoms, most of which are now recognized symptoms of myocardial ischemia or infarction [47, 48]. In general, military combatants are at heightened CVD risk, and often display sympathetic hyperactivation, accompanied by elevated blood pressure and decreased HRV [49].

Mental stress, whether in military combat or civilian trauma, can provoke acute myocardial ischemia that may become chronic if PTSD develops. Often silent and not directly related to CAD [50, 51], this stress-induced myocardial ischemia is likely ascribable to coronary vasospasm or peripheral vasoconstriction and the resulting systemic hypertension. Sustained mental stress-induced myocardial ischemia may increase the risk of CAD in PTSD patients [51]. Hassan *et al*. [52] hypothesized that susceptibility to mental stress-induced myocardial ischemia may be genetically determined through a β_1_-adrenergic receptor polymorphism.

An extreme manifestation of mental stress-induced myocardial ischemia is Takotsubo syndrome (TS) or “broken heart syndrome” [15, 53], a cardiomyopathy which can be triggered even without significant CAD. This acute, transient, but repetitive disorder can cause sudden cardiac death, fatal and non-fatal myocardial infarction, cardiomyopathy, heart failure, stroke, arrhythmias, hypertension, and pulmonary embolism [54]. TS can be triggered by distressing news, e.g., a serious clinical diagnosis [55, 56] or severe depression [57]. Large increases in TS as well as in concurrent PTSD have been observed in survivors of severe earthquakes, even those without a history of CVD [54, 58].

The pathogenic mechanisms of myocardial injury in TS and PTSD share key steps. Acute stress elicits a sharp rise in catecholamines that act directly on the coronary circulation and myocardium to trigger transient left ventricular dysfunction [56, 57]. Patients may develop chest pain, sweating, palpitations, electrocardiographic changes suggestive of acute myocardial infarction, and even cardiogenic shock. An analysis of international registries showed that elevated myocardial proteins troponin I and N-terminal pro-brain natriuretic peptide are serum biomarkers that predict the development of CVD in both TS and PTSD patients [59].

Patients with serious diseases requiring surgical treatment often have PTSD and its sequelae. Such patients face a heightened risk of postoperative cardiovascular complications and protracted hospitalization [60, 61, 62, 63]. PTSD augurs prolonged post-operative recovery and hospital stay following cardiac surgery [15]. Moreover, severe stress-related conditions, including PTSD, can cause myocardial injury after non-cardiac surgery and may impose postoperative cardiovascular complications even in the absence of pre-operative CVD [64]. Myocardial injury after non-cardiac surgery is strongly associated with high mortality [65]. About half of cardiac deaths occur in patients without a history of cardiac disease [15], so identifying biomarkers that predict heart damage in PTSD patients undergoing noncardiac surgery is essential.

The impact of preexisting PTSD on post-surgical cardiac damage and recovery was recently investigated in mice [66]. Prior to non-cardiac surgery (laparotomy), these animals were exposed to predator stress, i.e., cat urine, a validated model of PTSD [25, 67]. After surgery, the PTSD mice had elevated serum corticosterone and high-sensitivity cardiac troponin I compared to non-stressed mice undergoing a similar laparotomy. The PTSD animals had significant post-surgical injury to cardiomyocytes consistent with ischemic disruption of myofibrils. Also, in the PTSD mice, myocardial glycogen was significantly depleted, and markers of oxidative stress were increased, while antioxidant defense was impaired. These studies showed that PTSD can promote myocardial injury after noncardiac surgery, and confirmed earlier findings that non-specific stress biomarkers, such as corticosterone and troponin, can predict this outcome [64].

Cardiac troponins, especially troponin I, are released exclusively by cardiomyocytes and thereby serve as highly specific cardiac injury biomarkers [65]. Troponin is released through the damaged membranes of injured and dead cardiomyocytes in response to conditions typically associated with severe stress and PTSD, including myocardial ischemia, tachycardia, inflammation, and high serum catecholamine concentrations. Even in hypoxic but non-infarcted myocardium, small cardiac troponin I fragments can cross the cardiomyocyte sarcolemma [65]. Thus, increased serum cardiac troponin I following mental stress can indicate myocardial ischemia [64].

Traditional assessments of heart function and pathology, including exercise tests, coronary angiography, nuclear imaging, and the coronary artery calcium score, characterize those individuals resistant to PTSD-associated cardiac disease. Such PTSD resilience has been demonstrated in experimental animals [25]. Rats pre-exposed to predator stress were assigned to PTSD-susceptible (i.e., high anxiety) and PTSD-resilient (i.e., low anxiety) groups based on elevated plus-maze anxiety testing. A forced swimming test demonstrated that the PTSD-susceptible rats were less exercise-tolerant than the PTSD-resilient rats, whose exercise tolerance did not differ from that of rats not exposed to predator stress. PTSD-susceptible rats showed electrocardiographic changes, e.g., prolonged QRS and QT intervals, which paralleled histo-morphological hallmarks of cardiomyocyte injury [25]. Anxiety associated with experimental PTSD was correlated with electrocardiographic alterations [68], i.e., prolonged QRS complexes that reflected slower propagation of ventricular depolarization [69]. Similar electrocardiographic alterations are associated with intraventricular conduction disorders in human heart failure and myocardial ischemia [69, 70]. PTSD-susceptible rats also had elongated QT intervals, indicating slower ventricular repolarization, which can also reflect cardiotoxicity of exogenous substances [69, 71]. Since only the PTSD-susceptible rats displayed these electrocardiographic findings, PTSD resilience was cardioprotective [25]. Myocardial histology of the rats with PTSD revealed hallmarks of ischemic injury commonly observed in early myocardial infarction [72], including loss of cross-striatal structure of myofibrils caused by I-disk destruction and merged A disks, focal disaggregation, myofibril lysis, and impaired contractility [25, 73, 74]. These changes are generally considered reversible by regeneration, but may become irreversible with prolonged stress [75].

Depletion of myocardial glycogen was another histological finding in the PTSD rats [25, 76]. Myocardial glycogen content was significantly higher in the PTSD-resilient vs. the PTSD-susceptible rats, and did not differ from the unstressed control rats [25, 76]. Since myocardial glycogen is a critical energy reserve that sustains cardiac function during exercise [77], these findings were concordant with exercise tolerance tests, which differentiated PTSD-susceptible from PTSD-resilient animals. Thus, exercise tolerance tests may distinguish PTSD susceptibility vs. resilience in humans. Collectively, research in rodent PTSD models has yielded indispensable insights on the mechanisms of PTSD resilience vs. susceptibility, and on the evolution of cardiac and vascular injury inflicted by chronic adrenergic and glucocorticoid excess.

## 4. Vascular Injury and Resilience in PTSD

**Fig. 1.**
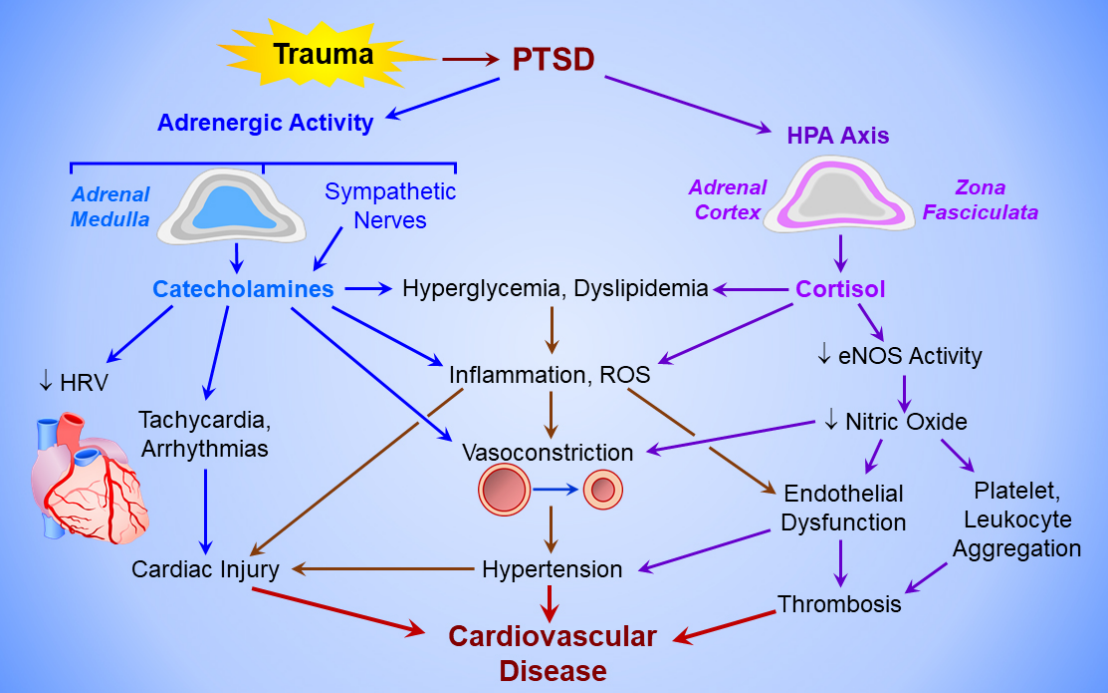
**Mechanisms of PTSD-induced cardiovascular disease**. Post-traumatic stress disorder (PTSD) activates the adrenergic system (upper left) to elicit catecholamine release from the adrenal medulla and sympathetic nerves, and the hypothalamo-pituitary-adrenal cortical (HPA) axis (upper right). The chronic hyperadrenergic state increases heart rate, decreases heart rate variability (HRV), triggers cardiac arrhythmias, and activates sympathetic vasoconstriction, producing hypertension and increasing left ventricular afterload. Catecholamines and cortisol from the adrenal medulla and cortex, respectively, provoke hyperglycemia and dyslipidemia, and induce inflammation and reactive oxygen species (ROS) formation, which injure the myocardium and vascular endothelium. Cortisol also inactivates endothelial nitric oxide synthase (eNOS), thereby disabling endothelium-dependent vasodilation and increasing coagulability of circulating platelets and leukocytes, producing a pro-thrombotic state. Cardiac injury, hypertension, and vascular thrombosis culminate in cardiovascular disease.

Chronic, PTSD-associated hyperactivation of the hypothalamic-pituitary-adrenal and sympatho-adrenomedullary axes and dysregulation of the autonomic nervous system [78, 79] elicit inflammation, endothelial dysfunction, hypercoagulability, and vascular hyperreactivity harmful to blood vessels (Fig. 1). Increased catecholamines and cortisol constrict arterioles, raise heart rate and blood pressure, and elevate risk of cardiac arrhythmias. These factors may trigger ischemic stroke or sudden cardiac death in acute PTSD, as in TS [79]. Chronic exposure to stress hormones can provoke sustained cerebral vasospasm, highly correlated with plasma concentrations of epinephrine and norepinephrine metabolites [78]. Importantly, PTSD is an independent risk factor for stroke. Incidence of ischemic stroke and transient ischemic attacks is threefold greater in PTSD patients [80], and stroke occurs at a significantly younger age in these patients [79].

Stroke and PTSD share multiple risk factors [24, 79, 80, 81, 82], e.g., female gender and black race are independently related to heightened risk of stroke. Modifiable risk and causative factors for stroke in PTSD include smoking, sedentary lifestyle, sleep disorders, unhealthy diet with ensuing metabolic syndrome and obesity, inflammation, and abnormal lipid profile. These risk factors and associated diseases, e.g., hypertension and type 2 diabetes, are strongly associated with PTSD and, thus, are considered predictive markers for stroke susceptibility in PTSD patients [30]. For example, catecholamine- and glucocorticoid-induced fatty acid release may produce dyslipidemia, another predictive biomarker of stroke [83]. Furthermore, any increase in total serum cholesterol corresponds to an increase in ischemic stroke incidence [84]. PTSD patients frequently have elevated inflammatory markers, specifically IL-6, TNF-α, and high-sensitivity C-reactive protein, and low HRV, which also predict susceptibility to stroke and its adverse outcomes [24]. PTSD is also associated, in both men [85] and women [86], with increased arterial wall stiffness, another independent predictor of cardiovascular morbidity and mortality [87].

As already noted, sympathetic activation in PTSD provokes cholesterol synthesis and fatty acid release from adipose triacylglycerol stores, culminating in abnormal serum lipid profile [81]. PTSD is positively associated with high total cholesterol, low-density lipoproteins, and triglycerides, and is inversely related to high-density lipoproteins; this serum lipid pattern may serve as a biomarker to assess PTSD-related cardiovascular risk. Atherosclerosis thickens and hardens the arterial wall and narrows the vascular lumen. Ahmadi *et al*. [88] showed coronary artery calcium score to be an accurate marker of overall atherosclerotic burden and the associated oxidative stress, endothelial dysfunction, plaque burden, obstructive CAD, and CVD in individuals with PTSD. Moreover, PTSD was associated with the severity of atherosclerosis and high mortality independent of age, gender, and other conventional risk factors. These results identified the coronary artery calcium score as a reliable biomarker of CVD susceptibility to PTSD.

A pivotal CVD risk factor, endothelial dysfunction with impaired endothelium-dependent vasodilation, is considered both a marker of and a powerful contributor to the pathogenesis of cardio- and cerebrovascular disease, including stroke [89]. While healthy endothelium regulates blood flow and blood pressure to maintain tissue perfusion and prevent hypertension, endothelial dysfunction is generally associated with impaired vasodilation, vasospasm, inflammation, vascular proliferation and fibrosis, and elevated prothrombotic factors. Stress-induced elevations of serum catecholamines and cortisol may damage the endothelium and accelerate atherosclerosis [89]. Patients with PTSD often have endothelial dysfunction that directly correlates with PTSD severity; moreover, endothelial dysfunction can predict PTSD vulnerability [87, 90, 91]. PTSD-susceptible individuals have blunted brachial artery blood flow velocity response to acute mental stress, indicating more intense vasoconstriction and endothelial dysfunction than in PTSD-resilient individuals [87].

Cerebrovascular endothelial function and cerebral blood flow were compared in experimental PTSD susceptible and resilient rats [92]. The PTSD-susceptible rats had pronounced endothelial dysfunction, evident as a vasoconstrictor response to a physiological vasodilator, acetylcholine. Consequently, basal cerebral blood flows in the PTSD-susceptible rats were appreciably less than those of PTSD-resilient rats. Cerebral blood flow correlated inversely with the anxiety index, suggesting that anxiety, which is elevated in PTSD, may indicate increased risk of cerebrovascular disorders.

The central mechanism of endothelial dysfunction is impaired basal and stimulated formation of nitric oxide, a powerful vasodilator, inhibitor of platelet and leukocyte aggregation, and anti-inflammatory agent [93]. Dysregulation of endothelial nitric oxide synthase (eNOS) likely contributes to endothelial dysfunction in PTSD. Acute and chronic stress, including PTSD, provoke oxidative stress, inflammation, and high glucocorticoid concentrations that collectively disrupt the regulation of coronary vascular nitric oxide formation [94, 95]. Experimental PTSD decreased eNOS content and activity, and loss of eNOS was associated with more pronounced impairment of basal cerebral blood flow and endothelial function in PTSD-susceptible rats [92].

PTSD can induce expression of procoagulant factors through stress-responsive hormones and thereby promote thrombosis, raising the risk of myocardial infarction, stroke, and other cardiovascular conditions related to blood hypercoagulation [79]. Clinical studies showed that PTSD induces thrombogenesis by increasing blood concentrations of factor VIII, von Willebrand factor, and fibrinogen, and by potentiating platelet aggregation. The decreased prothrombin time and activated partial thromboplastin time in individuals with PTSD reflect these prothrombotic processes [96, 97]. Platelet reactivity is increased in PTSD patients and correlates with the severity of PTSD symptoms. Moreover, PTSD severity parallels elevated plasma concentrations of factor VIII and fibrinogen [97]. These data are consistent with studies in PTSD-susceptible rats demonstrating reduced prothrombin time and activated partial thromboplastin time, and increased fibrinogen concentration and platelet aggregation [92]. Conversely, hemostatic parameters in PTSD-resilient rats did not differ from the non-stressed control rats.

Endothelial dysfunction is an important mechanism of stress-induced hypercoagulation (Fig. 1). Normally, endothelial nitric oxide limits platelet aggregation, but when nitric oxide production and/or bioavailability are impaired, the endothelium loses its anticoagulant and fibrinolytic properties [98]. Indeed, in PTSD-susceptible rats, the hemostatic phenotype was associated with endothelial dysfunction and reduced abundance of messenger RNA encoding eNOS [92]. Thus, parameters of blood coagulation are biomarkers that can predict cardiovascular susceptibility or resilience to PTSD.

Preservation of cerebral blood flow in PTSD-resilient rats was associated with increased cerebral dopamine concentrations [92]. Increased cerebral dopamine was associated with PTSD resilience, while PTSD risk was elevated in rats with low cerebral dopamine [99, 100]. This antiparallel dopamine-PTSD relationship is genetically determined [101]. In rats modeling stress-induced depression and anxiety, chronic exposure to unpredictable stress depleted hippocampal dopamine, induced pro-inflammatory TNF-α, provoked neuronal apoptosis and blunted neuronal genesis and differentiation [102]. In addition, dopamine exerts a direct vasodilatory effect by stimulating eNOS expression [103]. These findings in rats paralleled those in war veterans with PTSD, where viewing a video with trauma-related footage provoked an acute decline in the dopamine metabolite homovanillic acid in the cerebrospinal fluid [104]. An increase in cerebral dopamine may represent a marker for PTSD resilience, and raises the possibility that pharmacological activation of dopaminergic neurotransmission may effectively attenuate PTSD.

In summary, increased PTSD susceptibility or the presence of PTSD is associated with stress-induced vulnerability of blood vessels to damage and dysfunction. Increased total cholesterol, dyslipidemia, arterial stiffness, high blood pressure, and impaired endothelial function, along with reduced eNOS and dopamine, may serve as important markers and predictors of PTSD-related vascular damage.

## 5. Mechanisms of Cardiovascular Damage and Resilience in PTSD

### 5.1 Hypothalamic-Pituitary-Adrenal Axis, Sympathetic Nervous System, and Autonomic Imbalance

The sympathetic nervous system (SNS) and the hypothalamic-pituitary-adrenal (HPA) axis are major stress response systems implicated in cardiovascular damage in PTSD patients (Fig. 1) [31, 105]. The SNS responds to stress first by releasing epinephrine and norepinephrine from the adrenal medulla and adrenergic nerve endings, respectively. The slower HPA hormonal cascade triggers more gradual release of cortisol from the adrenal cortex. Activation of the SNS and the HPA system is a protective reaction to stress, but sustained and augmented increases in catecholamines and cortisol in PTSD patients are detrimental to the heart and blood vessels [31].

Negative feedback mediated by glucocorticoid and mineralocorticoid receptors determines the duration of the stress response [19, 31]. PTSD is generally associated with hypertension and increased resting heart rate [105]. Post-stress prolongation of biomarkers of SNS and HPA activation, e.g., elevated blood pressure and heart rate, directly correlates with PTSD severity [106]. When patients with PTSD experience new stress, an exaggerated increase in blood pressure indicates heightened risk of hypertension and other cardiovascular diseases [107]. Dysregulation of the SNS and HPA axis correlates with PTSD severity and plays a pivotal role in acute and chronic stress injury of the cardiovascular system in PTSD patients [31, 32].

Decreased parasympathetic nervous system activity accompanies the increased SNS activity in PTSD patients, and the resultant autonomic imbalance contributes to CVD development [108]. Analysis of HRV, a biomarker of parasympathetic nervous system activity and stress resilience [109, 110], assesses autonomic dysfunction. Low HRV predicts PTSD vulnerability; thus, low pre-deployment HRV in US Marines significantly predicted post-deployment PTSD [111]. On the other hand, normal pre-stress HRV could be considered a marker of PTSD resilience, and likely is present in many individuals who experience traumatic stress yet do not develop PTSD.

The parasympathetic activity indicator HRV, and serum cortisol or the cortisol/dehydroepiandrosterone ratio as measures of HPA activity, are strong positive and negative predictors, respectively, of psychological resilience. Lau *et al*. [112] reported that the pre-stress cortisol/dehydroepiandrosterone ratio varied inversely with stress resilience, while high baseline HRV and recovery of the cortisol/dehydroepiandrosterone ratio after stress were positively associated with resilience. Thus, HRV and the cortisol/dehydroepiandrosterone ratio may serve as biomarkers for PTSD resilience. Decreased parasympathetic control is a known contributor to the risk of major adverse cardiovascular events (MACE). Thus, decreased HRV represents a reliable biomarker for both PTSD resilience/susceptibility and MACE in patients with PTSD [18].

Stress activation of HPA causes adrenocortical release of glucocorticoids (cortisol in humans, corticosterone in rodents), while PTSD development is associated with decreased glucocorticoid concentrations in blood, saliva, and urine [113]. Furthermore, the lower the cortisol level, the higher the likelihood of PTSD development. Accordingly, hydrocortisone treatment reduces the risk of PTSD [114]. PTSD-resilient rats showed little or no post-stress decrease in corticosterone, while PTSD-susceptible animals showed a pronounced decrease in corticosterone, which persisted at least one month after predator stress [115, 116]. The negative correlations between the anxiety index vs. serum corticosterone concentrations and adrenal deoxycorticosterone contents confirmed the role of glucocorticoids in PTSD resilience. These data are concordant with an earlier study in police officers, where a blunted or absent cortisol response to psychological stress prospectively predicted low PTSD resilience [117]. The post-stress drop of corticosterone with PTSD may be ascribable, at least partially, to damage of glucocorticoid-secreting cells in the adrenal zona fasciculata. In poorly resilient rats, accumulation of injured and degenerating cells accompanied pronounced thinning of the zona fasciculata [118, 119]. Moreover, correlation of zona fasciculata thinning with the anxiety index underscored the pivotal role of adrenocortical function in PTSD resilience [119, 120].

### 5.2 Systemic Inflammation and Oxidative Stress

Serum pro-inflammatory cytokines are increased and anti-inflammatory cytokines are decreased in PTSD patients [121, 122]. Indeed, inflammation is so tightly associated with PTSD that some researchers have suggested that PTSD is essentially an immunological and inflammatory disorder that predisposes to other inflammatory conditions, e.g., cardiovascular and metabolic diseases [121]. Even exposure to severe stress not directly trigger PTSD can provoke inflammatory responses with increased blood concentrations of proinflammatory markers [123]. Moreover, inflammatory conditions are PTSD risk factors [124], while anti-inflammatory therapies have proven efficacious for PTSD treatment [122]. Activation of the HPA axis triggers the low-grade inflammation observed in PTSD [31]. Circulating catecholamines stimulate the bone marrow, spleen, lung, and lymph nodes to release immune cells, which migrate to target tissues, where they activate inflammation.

Stress-induced changes in inflammatory markers are significantly associated with pre-stress PTSD symptoms and predict the post-stress risk and onset of PTSD. In Special Forces personnel, Bennett *et al*. [125] reported that the pre- to post-deployment increases in serum C-reactive protein and IL-6 correlated with subthreshold PTSD symptoms and were predictive biomarkers for PTSD susceptibility. Eraly *et al*. [126] showed that pre-deployment plasma concentrations of high-sensitivity C-reactive protein in US Marines correlated with Clinician-Administered PTSD assessment scale after their deployment in a combat zone.

A meta-analysis of 54 studies demonstrated markedly elevated serum C-reactive protein, IL-6, and TNF-α concentrations, and more modest increases in IL-1β in PTSD patients [33]. Another meta-analysis [127] found increased concentrations of IL-1 and IL-6, interferon γ, and TNF-α in PTSD patients, and defined patterns of inflammatory markers associated with PTSD. IL-1 and IL-6 concentrations were identified as biomarkers of PTSD duration and severity, respectively.

The circulating neutrophil-lymphocyte ratio and the systemic immune inflammation index are new composite markers for evaluating subclinical systemic inflammation in psychiatric patients, including those with PTSD [128]. Both the neutrophil-lymphocyte ratio and systemic immune inflammation index were elevated in patients with vs. without PTSD. High neutrophil-lymphocyte ratio also independently predicted adverse outcomes in acute heart failure [129] and mortality in CAD [130] and ST- and non-ST-elevation myocardial infarction [131]. Systemic immune inflammation index is an immunothrombosis biomarker [128] and a predictor of metabolic syndrome, hyperglycemia, and hypertension [132] and severity of CAD and acute ischemic stroke [133, 134]. Elevated in both PTSD and MACE, serum high-sensitivity C-reactive protein is a likely biomarker of the inflammatory mechanisms linking PTSD with increased risk of MACE [18]. Serum concentrations of the inflammatory biomarkers IL-6, TNF-α, and high-sensitivity C-reactive protein were inversely associated with self-reported resilience and quality of life in women with PTSD [135].

The close relationship between PTSD and systemic inflammation suggests anti- vs. pro-inflammatory cytokine balance may serve as a PTSD resilience biomarker [136]. Indeed, PTSD resilience was associated with increased serum levels of anti-inflammatory IL-4 and IL-10 and decreased proinflammatory IL-12 in Norwegian navy cadets undergoing a stressful military field exercise [137]. Similar data were obtained in animal experiments [25], where PTSD-resilient rats had lower plasma and myocardial concentrations of the proinflammatory cytokine, IL-6, and higher concentrations of the anti-inflammatory cytokine, IL-4, vs. PTSD-susceptible rats.

Thus, inflammatory biomarkers are simultaneously indicators, causative factors, and predictors of PTSD. Consequently, the cytokine response to stress apparently largely determines cardiovascular resilience to PTSD.

Inflammation and oxidative stress frequently occur in parallel and even activate each other reciprocally [138]. Overproduction of proinflammatory cytokines in PTSD provokes reactive oxygen species (ROS) formation, which intensifies as PTSD progresses [139]. Oxidative stress is a central mechanism of damage to the heart and vasculature [19]. In blood vessels, ROS stimulates smooth muscle cell growth and proliferation, resulting in maladaptive remodeling. ROS also uncouples eNOS by depleting the eNOS cofactor tetrahydrobiopterin, thereby reducing NO bioavailability [87] and making eNOS a ROS-generator [140]. At the same time, low PTSD resilience is associated with endothelial dysfunction [87, 92], an early indicator of diminished vascular capacity to respond to the metabolic demands of the cardiovascular system, and of increased risk of atherosclerosis and myocardial damage [141].

Lipid peroxidation assessed by serum malondialdehyde was more intense in earthquake survivors who developed PTSD than in those who did not [142]. The concentrations of oxidative stress biomarkers, including ROS products, conjugated dienes, and carbonylated proteins, were significantly higher in the myocardium and plasma of PTSD-susceptible vs. PTSD-resilient rats [25]. Oxidative stress-induced damage is determined by an imbalance, observed in PTSD [138], between ROS production and the activity of endogenous antioxidant systems, mainly the enzymes catalase, glutathione peroxidase, and superoxide dismutase. Normally, endogenous antioxidant systems are activated in response to a moderate increase in ROS production, but antioxidants often are depleted in PTSD [142, 143]. Weakened antioxidant defense is a major determinant of cardiovascular susceptibility to PTSD [144]. The possibility that antioxidant supplements could at least partially mitigate CVD in PTSD patients has not been addressed, but merits attention.

## 6. Can Effective PTSD Treatment Ameliorate the Cardiovascular Comorbidities?

Although responsive to clinical interventions, PTSD is difficult to cure, and many patients require monitoring and treatment throughout their lifetime. Clinical interventions for PTSD center on trauma-based psychotherapy and pharmacological interventions. According to the 2025 Clinical Practice Guidelines of the American Psychological Association [145], cognitive behavioral and cognitive processing therapy, trauma-based cognitive behavioral therapy, and prolonged exposure to triggers and memories are first-line recommendations for PTSD, while cognitive therapy, eye movement desensitization-reprocessing, and narrative exposure therapy are second-line recommendations for which evidence of efficacy is less robust. Pharmacologically, selective serotonin reuptake inhibitors and the serotonin-norepinephrine reuptake inhibitor, venlafaxine, have proven moderately efficacious for PTSD-associated depression and anxiety [146], although only the serotonin reuptake inhibitors, sertraline and paroxetine, are approved by the U.S. Food and Drug Administration for PTSD treatment [145]. The α_1_-adrenergic antagonist prazosin and non-selective β-adrenergic antagonist propranolol have proven efficacious as off-label treatment for managing PTSD symptoms, including nightmares and arousal [147, 148, 149].

The extensive evidence implicating PTSD in a host of CVD comorbidities raises an important question: could effective PTSD treatment achieve partial or complete reversal of PTSD’s cardiovascular sequelae? A growing body of clinical evidence supports this possibility, although not unequivocally. Bourassa *et al*. [150] conducted a comprehensive meta-analysis of clinical trials examining cardiovascular responses to PTSD treatment. Eleven of the 12 studies using cognitive behavioral therapy for PTSD reported some reduction in cardiovascular reactivity. Two high-quality studies with substantial numbers of PTSD patients identified statistically significant, albeit modest, reductions in cardiovascular reactivity to trauma-specific stressors in patients receiving cognitive behavioral therapy [151]. Four studies demonstrated statistically significant associations of PTSD symptom relief and improved HRV, but three did not. Van den Berk Clark *et al*. [152]’s meta-analysis of efficacious PTSD treatments revealed that cognitive behavioral therapy and Selective Serotonin Reuptake Inhibitors(SSRIs) lowered arterial blood pressure, prolonged exposure therapy lowered resting heart rate, and cognitive behavioral therapy and prolonged exposure lowered HRV. Both meta-analyses identified an unmet need for statistically robust, placebo-controlled trials examining the impact on CVD of trial-specific first-line PTSD interventions [150, 151]. Although most of the analyzed trials achieved parallel, partial reductions in PTSD and CVD risk, the post-treatment persistence of the cardiovascular benefits remains unclear and is an important focus of clinical research. Moreover, the incomplete resolution of CVD suggests combining psychotherapeutic PTSD interventions with CVD-focused medications, e.g., anti-adrenergic or anti-inflammatory agents, may prove more efficacious than monotherapies against CVD.

Recent studies have evaluated physical exercise as a standalone or adjuvant intervention for PTSD [4]. Several clinical trials of aerobic exercise in adults with PTSD demonstrated reductions in hyperarousal, avoidance, sleep disturbances, and depression following 2–12-week aerobic exercise programs consisting of cycling, brisk walking, jogging and/or resistance exercise, with similar benefits regardless of exercise modality [153, 154, 155, 156]. However, none of these studies examined CVD-specific biomarkers as primary or secondary endpoints. Consequently, the capacity of exercise programs to augment CVD resilience in individuals with PTSD has yet to be evaluated.

## 7. Summary and Future Directions

PTSD, a devastating psychiatric condition affecting nearly half a billion persons worldwide, elicits a complex neuroendocrine and inflammatory cascade with profound, adverse impact on the heart and cardiovascular system. Although the mechanisms making a trauma victim vulnerable vs. resilient to PTSD are unclear, several chemical and functional PTSD biomarkers have emerged, including circulating inflammatory mediators, cardiac troponins, malondialdehyde, protein carbonyls, conjugated dienes, catecholamines and glucocorticoids, increased resting heart rate and blood pressure, altered HRV, prolonged QRS complexes and QT intervals, vascular endothelial dysfunction, platelet and leukocyte aggregation, and increased neutrophil-lymphocyte ratio and systemic immune inflammation index. Further understanding of the maladaptive processes linking PTSD and cardiovascular dysfunction/disease will help to identify potential treatment targets, raising the possibility that combining PTSD psychotherapies with pharmacological interventions targeting cardiovascular injury mechanisms may be particularly efficacious. Preclinical research on mechanisms linking PTSD and CVD, and expanded, high-quality clinical trials of behavioral and pharmacological interventions, potentially will bolster the therapeutic armamentarium against these daunting, interrelated disorders.

Preclinical and clinical studies discussed in this review have identified several candidate biomarkers of cardiovascular susceptibility to vs. resilience against PTSD (Table 1, Ref. [16, 17, 24, 25, 27, 32, 33, 40, 44, 45, 51, 52, 59, 64, 66, 84, 87, 88, 90, 91, 92, 96, 97, 106, 107, 109, 110, 112, 115, 116, 117, 124, 125, 126, 127, 128, 135, 137, 142]). These biomarkers may be categorized as (1) physiological (e.g., HRV, arterial blood pressure responses to stressors, arterial pulse-wave velocity, flow-mediated vasodilation, baroreflex sensitivity); (2) genetic (e.g., polymorphisms of β-adrenoceptors, glucocorticoid receptors, ion channels, synaptic structural proteins, endocrine and immune regulators, neurodevelopmental factors, type 2 diabetes risk genes, innate immunity and inflammatory mediators); (3) hemostatic/thrombotic (circulating clotting factors, fibrinogen, prothrombin and activated thromboplastin times, time, platelet aggregation); (4) neuroendocrine (e.g., catecholamines, catecholamine metabolites, cortisol, dehydroepiandrosterone (DHEA)); (5) metabolic comorbidities (dyslipidemia, type 2 diabetes mellitus); (6) oxidative-inflammatory (malondialdehyde, inflammatory cytokines, C-reactive protein, neutrophil-lymphocyte ratio). Many of these biomarkers are measured routinely. Wearable blood pressure and HRV monitors are commercially available for home use. Blood pressures are taken at every patient encounter. Serum lipid profiles, glycemic index, hemostatic factors, and thrombosis are measured readily by diagnostic laboratories, as are catecholamines in urine samples and glucocorticoids in saliva. On the other hand, measurements of some biomarkers, e.g., pro- and anti-inflammatory cytokines, oxidative stress biomarkers, and genetic polymorphisms require more sophisticated analyses and equipment.

**Table 1.** **Experimental and clinical studies of PTSD-associated cardiovascular risk biomarkers**.

Author(s)	Ref.*	Study details	Biomarkers
**Studies in experimental animal models of PTSD**			
Manukhina *et al*., 2021	[25]	Experimental study (rats)	Exercise tolerance, corticosterone, QRS and QT intervals (electrocardiogram), interleukin (IL)-4, IL-6, conjugated dienes, and carbonylated proteins
Kondashevskaya *et al*., 2024	[66]	Preclinical (mice)	Corticosterone, high-sensitivity cardiac troponin I (hs-cTnI)
Kondashevskaya *et al*., 2022	[92]	Experimental study (rats)	Activated partial thromboplastin time (aPTT), cerebral endothelial dysfunction, dopamine, endothelial nitric oxide synthase mRNA, platelet activation, fibrinogen, prothrombin time (PTT)
Tseilikman *et al*., 2022	[115]	Experimental study (rats)	Corticosterone
Tseilikman *et al*., 2019	[116]	Experimental study (rats)	Corticosterone
**Studies in human subjects and patients**			
Hargrave *et al*., 2022	[16]	Narrative review of clinical literature	C-reactive protein (CRP), IL-6, tumor necrosis factor-alpha (TNF-α), modifiable lifestyle factors (sedentary lifestyle, unhealthy diet, smoking, alcohol or drug abuse)
Khalil *et al*., 2025	[17]	84,343 subjects in the Massachusetts General Brigham Biobank	Cardiovascular disease risk factors (hypertension, hyperlipidemia, type 2 diabetes mellitus, heart rate variability (HRV), CRP)
Nanavati *et al*., 2023	[24]	Review and meta-analysis of 8 cohort studies (3,738,222 subjects) and 3 cross-sectional studies (5168 subjects)	Lifestyle factors (smoking, sedentary lifestyle, sleep disorders, unhealthy diet, metabolic syndrome, obesity, abnormal lipid profile, type 2 diabetes mellitus, hypertension), IL-6, TNF-α, hs-CRP, HRV
Haag *et al*., 2019	[27]	76 child and adolescent trauma survivors	HRV
Fonkoue *et al*., 2020	[32]	Military veterans: 28 with severe PTSD, 16 with moderate PTSD, 26 without PTSD	Combined inflammatory score (E-selectin, TNF-α, IL-1β, IL-2, IL-6, IL-1-receptor antagonist, IL-6 receptor, interferon γ, monocyte chemoattractant protein-1, TNF-receptor II, intercellular adhesion molecule-1), HRV, baroreflex sensitivity
Peruzzolo *et al*., 2022	[33]	Meta-analysis: 54 clinical studies (8394 subjects)	CRP, IL-6, IL-1β, TNF-α
Meinhausen *et al*., 2022	[40]	Narrative review of clinical literature	Sleep disorders
Nievergelt *et al*., 2024	[44]	Meta-analysis of genome-wide association studies (150,760 cases)	Genetic markers (e.g., ion channels, synaptic structural proteins, endocrine and immune regulators, neurodevelopmental factors)
Pollard *et al*., 2016	[45]	Meta-analysis: 106 studies of 87 candidate genes (83,463 subjects)	Genetic markers (e.g., glucocorticoid receptors, type 2 diabetes risk genes, innate immunity, and inflammatory mediators)
Mehta *et al*., 2022	[51]	Clinical literature review	hs-cTnI: serum marker of mental stress-induced myocardial ischemia (MSIMI)
Hassan *et al*., 2008	[52]	Genetic study: 148 patients with coronary artery disease (CAD)	β_1_-adrenergic receptor polymorphisms (genetic MSIMI risk factors)
Song *et al*., 2012	[59]	Genetic study: 103 patients in the Takotsubo cardiomyopathy registry	β_1_-adrenergic receptor polymorphisms (genetic MSIMI risk factors)
Clerico *et al*., 2022	[64]	Clinical literature review: cardiac injury biomarkers	Brain natriuretic peptide/N-terminal pro-brain natriuretic peptide ratio, hs-cTnI
Heydari *et al*., 2025	[84]	Meta-analysis: 36 studies (871,447 subjects)	Dyslipidemia, fatty acids, total cholesterol
Tahsin *et al*., 2023	[87]	Clinical study: 60 healthy young adult women	Arterial stiffness (pulse-wave velocity), endothelial dysfunction (Framingham reactive hyperemia index)
Ahmadi *et al*., 2011	[88]	Clinical study: veterans with (*n* = 88) or without (*n* = 549) PTSD	Coronary artery calcium score
von Känel *et al*., 2008	[90]	Clinical study: 14 PTSD patients, 14 controls	Markers of endothelial dysfunction (soluble tissue factor, von Willebrand factor)
Grenon *et al*., 2016	[91]	Clinical study: veterans with (*n* = 67) or without (*n* = 147) PTSD	Endothelial dysfunction (flow-mediated vasodilation)
Sandrini *et al*., 2020	[96]	Clinical literature review	Platelet activation, factor VIII, von Willebrand factor, fibrinogen, PTT, aPTT
Austin *et al*., 2013	[97]	Clinical literature review	Soluble tissue factor, platelet activation, factor VIII, von Willebrand factor, fibrinogen, D-dimer, PTT, aPTT
Walker *et al*., 2017	[106]	Clinical literature review	Post-stress cardiovascular recovery, HRV, dehydroepiandrosterone (DHEA), cortisol/DHEA ratio, IL-1, IL-6, interferon γ, TNF-α
Yoo *et al*., 2020	[107]	Clinical study: 14 women with PTSD, 14 controls	Post-stress BP response
Agorastos *et al*., 2023	[109]	Clinical literature review	HRV
Schneider and Schwerdtfeger, 2020	[110]	Meta-analysis: 43 studies (5481 patients)	HRV
Lau *et al*., 2021	[112]	Clinical study: 107 healthy young adults	HRV, cortisol/DHEA ratio
Galatzer-Levy *et al*., 2014	[117]	Clinical study: 234 urban police officers	Cortisol
Ogłodek, 2022	[124]	Clinical study: 460 subjects with depression and PTSD	IL-1β, IL-4, IL-8, IL-10
Bennett *et al*., 2024	[125]	Clinical study (special forces personnel)	CRP, IL-6
Eraly *et al*., 2014	[126]	Clinical study: 2555 male marines	CRP
Passos *et al*., 2015	[127]	Meta-analysis: 20 studies (1348 subjects)	IL-1, IL-6, interferon γ, TNF-α
Islam *et al*., 2024	[128]	Clinical literature review	Neutrophil-lymphocyte ratio, systemic immune inflammation index
Imai *et al*., 2019	[135]	Clinical study: 56 women with PTSD, 73 controls	IL-6, hs-CRP
Sandvik *et al*., 2013	[137]	Clinical study: 21 navy cadets	IL-6, IL-12, IL-4, IL-10, neuropeptide Y
Atli *et al*., 2016	[142]	Clinical study: 63 earthquake survivors, 38 controls	Malondialdehyde

*Citation numbers as listed in References. Abbreviations: aPTT, activated partial thromboplastin time; CRP, C-reactive protein; DHEA, dehydroepiandrosterone; HRV, heart rate variability; hs-CRP, high sensitivity CRP; hs-cTnI, high sensitivity cardiac troponin I; IL, interleukin; MSIMI, mental stress induced myocardial ischemia; PTT, prothrombin time; TNF-α, tumor necrosis factor-alpha.

Although the most sensitive or reliable biomarkers of cardiovascular PTSD susceptibility vs. resilience are not yet established, there are compelling reasons to consider HRV as a potential frontline PTSD-CVD biomarker. As a readily measured, noninvasive index of autonomic function, HRV is well suited for monitoring parasympathetic-sympathetic balance in individuals with PTSD or those at risk of PTSD (see Table 1). HRV is responsive to physical exercise training, an intervention that has proven efficacious for PTSD treatment. Indeed, meta-analyses of randomized controlled clinical trials demonstrated aerobic exercise training programs increased HRV and cardiac parasympathetic control in healthy adults [157] and in patients with various CVD-related conditions including hypertension [158], postural orthostatic tachycardia syndrome [159], type 2 diabetes mellitus [160], chronic kidney disease [161], or post-coronary artery bypass surgery [162]. Importantly, HRV is a heart-specific variable unlikely to be confounded by noncardiac PTSD comorbidities. On that basis, HRV merits attention as a potential biomarker to evaluate CVD resilience vs. susceptibility in individuals with PTSD, and monitor the impact of aerobic exercise and other PTSD treatments on the cardiovascular system.
